# An ethnobotanical survey of medicinal plants used in the eastern highlands of Papua New Guinea

**DOI:** 10.1186/1746-4269-8-47

**Published:** 2012-12-18

**Authors:** Ronald Y Jorim, Seva Korape, Wauwa Legu, Michael Koch, Louis R Barrows, Teatulohi K Matainaho, Prem P Rai

**Affiliations:** 1 University of Papua New Guinea, School of Medicine and Health Sciences, PO Box 5623, Boroko, NCD, Papua New Guinea; 2 Department of Pharmacology and Toxicology, University of Utah, 30 S. 2000 E, Salt Lake City, Utah 84112, USA

**Keywords:** Papua New Guinea, Eastern Highlands, Medicinal Plants, Obura-Wonenara, Unggai-Bena, Okapa

## Abstract

**Background:**

The Eastern Highlands area of Papua New Guinea (PNG) has a rich tradition of medicinal plant use. However, rapid modernization is resulting in the loss of independent language traditions and consequently a loss of individuals knowledgeable in medicinal plant use. This report represents a program to document and preserve traditional knowledge concerning medicinal plant use in PNG. This report documents and compares traditional plant use in the Eastern Highlands districts of Unggai-Bena, Okapa, and Obura-Wonenara, and puts these new records in context of previously documented PNG medicinal plant use.

**Methods:**

This manuscript is an annotated combination of Traditional Medicines survey reports generated by UPNG trainees using a survey questionnaire titled “Information sheet on traditional herbal reparations and medicinal plants of PNG”. The Traditional Medicines survey project is supported by WHO, US NIH and PNG governmental health care initiatives and funding.

**Results:**

Overall, after “poisoning” (synonymous with *“magic”*) the most commonly recorded ailments addressed by medicinal plant use were pain, gynecological disease, gastrointestinal maladies, anemia or malnutrition and malaria. However, the recorded indications for plant use varied widely amongst the different survey locations. Unlike many areas of PNG, mixing of ingredients was the most common mode of preparation recorded, except for two areas where the consumption of fresh plant material was more common. Throughout the Eastern Highlands oral administration was most common, with topical application second. Overall, leaves were most commonly used in the preparations of the healers interviewed, followed by bark and stems. Several new medicinal uses of plants were also documented.

**Conclusions:**

Collaboration between the WHO, UPNG and the PNG Department of Health initiated Traditional Medicine survey program in order to preserve traditional knowledge concerning medicinal plant use in PNG. This effort promotes integration of effective and accessible traditional practices with Western protocols. The Traditional Medicine surveys are particularly important because, in the absence of the clinical validation, the documentation of the consistent use of a given plant for specific indication by a large number of herbalists, across a wide range of ethnic traditions, maybe considered as a positive criterion for the promulgation of said use amongst PNG’s recently formed traditional healer associations.

## Background

Rural communities in Papua New Guinea (PNG) rely on a tradition of plant use for health needs [1]. PNG has at least 800 ethnic traditions characterized by distinct languages scattered mostly in rural hamlets across a geographically segregated mountainous country of 462,840 km^2 ^[2,3], of which the Eastern Highlands Province occupies 11,200 km^2^. This geology has also resulted in extraordinary biological diversity estimated to be greater than 5% of the global total including an estimated 15,000 to 20,000 vascular plants, approximately 60% of which are endemic [4,5]. Human settlement has existed from at least 40,000 years BP on the north coast (Houn Peninsula) of PNG. More recently, human occupation of the Ivane Valley in the PNG Highlands has been dated to 49,000 to 44,000 years ago [6]. Of PNG’s approximately 6.2 million people about 430,000 reside in the Eastern Highlands [7]. This extended habitation of diverse environs has led to a rich and varied practice of medicinal plant use [8].

Reports from Western contact as early as the 1800s detailed the use of medicinal plants by PNG people to treat various maladies [9]. This Western perception of the cultural tradition of medicinal plant use, however, has been disparaged in some literature because often the curing properties of the plants are conceived by the users as magical [10]. This was the case in PNG Highlands where, until recently, non-western concepts of illness etiology predominated. Nevertheless, as in most of PNG, it is the current practice in the Highlands to use different plants to treat various symptoms regardless of whether the cause of the symptoms is conceived of as mystical or somatic.

The fact that a sizable majority of the PNG population relies on medicinal plants and traditional practitioners for health care has been formally recognized by the national government [1]. The 2001–2010 PNG National Health Plan promoted collaboration between the World Health Organization (WHO) and the University of Papua New Guinea (UPNG) to assist in the development of traditional medicines in the country. A traditional medicines survey instrument was developed using WHO guidelines and with vetting from Western Pacific WHO regional officers. In 2001 the UPNG Traditional Medicines surveys were initiated with endorsements from the UPNG School of Medicine and Health Sciences Research and Ethics Committee and the Medical Research and Advisory Committee of the PNG Department of Health. A proprietary database for traditional medicines was also established, which is maintained at UPNG [11], and now serves as a national resource as the government seeks to move validated and safe herbal remedies into the national health care formulary [1].

It is widely recognized that the traditional use of medicinal plants constitutes an important information reservoir that is threatened by on-going development and Westernization. This cultural reservoir of knowledge has been empirically tested and adopted through millennia of trial and error, but prior to the UPNG Traditional Medicines surveys, three of which are reported here, there was no national effort to preserve such knowledge. The documentation of medicinal plants in PNG has been haphazard and the accrued knowledge has not been widely disseminated internationally. Furthermore, the corresponding pharmacological validation of PNG medicinal plant use has not been systematically studied. We estimate that historically some 800 PNG plants species have been described in the literature for treatment of various ailments, but this represents only a fraction of the total number of plants actually utilized.

We present here a survey of traditional healers and report their current uses of medicinal plants from three distinct ethnic regions of the Eastern Highlands. The objective is to document and preserve knowledge of medicinal plant use in the Eastern Highlands of PNG, to use this activity as a scientific and culturally affirmative training exercise for senior UPNG students, and to put the recorded plant use into the PNG context by comparison with archived literature concerning medicinal plant use in PNG. This activity has identified many species used medicinally in the Eastern Highlands that are also used elsewhere in PNG, and a few species for which there are rare or no literature reports of medicinal use.

## Methods

The Traditional Medicine survey program at UPNG provides an effective training exercise for select senior bachelor of pharmacy students. The Traditional Medicines Database currently contains cultural plant use data from over 34 Local Level Governments (LLGs) in PNG. Students are instructed on plant identification and preservation, herbal medicine use, and how to administer the survey instrument titled “Information sheet on traditional herbal preparations and medicinal plants of Papua New Guinea”. This survey instrument questionnaire is designed to facilitate semi-structured face-to-face interviews with healers. The interaction is directed at recording new data concerning the medicinal uses of plants and the related cultural traditions, and includes field work for the collection of plant vouchers. The students are supported to travel to their home districts to conduct the surveys amid “wantok” communities. Wantok implies more than a common language in PNG, encompassing a meaning of extended kinship as well. Students meet first with elders, village heads, ward councilors, etc., in the study communities to gather names of healers before contacting them with the request for interviews. The principal requirement being that the healers are recognized in the community to have knowledge and skills of providing herbal treatments, that they are active in the practice and are willing to share their knowledge. The interviews are usually conducted in Tok Pisin or Tok Ples (local dialects) because facility with English is not uniform amongst the interviewees and it is important to communicate as clearly as possible.

Samples of the plants useful for identification (flowers, fruits or nuts, twigs with leaves) in addition to the parts used medicinally were harvested, dried and compressed in newspapers. A copy of the survey questionnaire is provided in supplementary information Additional file 1. Newspapers were changed daily until they remained dry after compression. Pressed plant samples, plant photographs and descriptions were assigned a voucher numbers and deposited at the UPNG Herbarium for identification and reference purposes [8]. The data concerning plant use are written up under supervision into student authored reports and the plant information is entered into the Traditional Medicines Database, which contains the combined reports generated by a decade’s work in this endeavor. It is the student reports that provide the base information for this current report.

Guidelines regulating accession of the database have been developed at UPNG in order to recognize and trace the traditional knowledge and intellectual property of the source communities. The guidelines operate under the current UPNG benefit sharing model, which is applicable to many areas of natural products research and which includes guidelines concerning intellectual property rights and benefits sharing that has been approved by the PNG government. The proprietary UPNG Traditional Medicines Database records, in addition to plant medicinal use, information concerning source individuals and communities in order to recognize and trace the traditional knowledge intellectual property.

**Figure 1:**
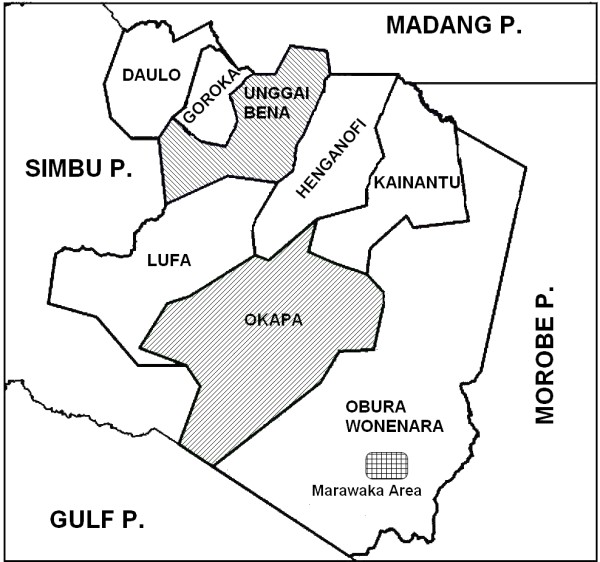
Map of Eastern Highlands Province of Papua New Guinea showing Local Level Governments (LLG) where studies were carried out.

The three student reports compiled here detail medicinal plant use from three separate regions and several distinguishable language and dialect groups in the Eastern Highlands province of PNG (Figure 1). The Eastern Highlands areas surveyed encompass Ipma-Baruya language groups of the Marawaka area of the Yeila Rural Local Level Government (LLG), Obura-Wonenara electorate; the Bena, Gahuku and Siane language groups of the Unggai-Bena LLG; and the Fore/Keyagana/Kimi language groups of the Okapa LLG [12]. Forty five practitioners were interviewed for this work: three from Ande village, 16 from Jomuru village, one from Marawaka station, five from Wauko village, one from Boike village, one from Gawol village, one from Kam’mwa village, and one from Kwaksiolo village in the Marawaka/South Obura Wonenara LLG area; two from Numurapoka village, one from Ginipauka village, one from Masiga, two from Orumba village, and two from Sekagu village from the Unggai Benna LLG area; four from the Haga villages, two from Yavanita village and two from Amusa village in the Okapa LLG of the Eastern Highlands.

## Results and discussion

### Dataset and diseases

A total of 60 specimens were described from the Marawaka area, 76 from the Okapa LLG; and 77 in the Unggai-Bena LLG. Two plants specimens each in the Marawaka and Okapa constituencies only served as additives or spices: *Piper gibbilimbum* and a *Saccharum* sp. to mask bitter tastes of herbal preparations; *Saccharum* species are commonly used as a salty preservative. In the Kayagana language area, *Rungia klossii* and *Zingiber officinale* are the two species that are routinely added to various medicinal preparations. The addition of pork to concoctions is frequent in all areas studied, but especially in the Ungaai-Bena LLG, where the practice was recorded as nearly universal. Pork is a high protein food and cultural icon in the Highlands.

The total number of diseases and symptoms listed in this report is 220, with several species recorded as having more than one use. Disease descriptions have been sorted into 14 general categories as shown in Table 1. Overall, by far the largest category of plant use (n = 45 [20.5%]) was “*magic”*, synonymously referred to here as “poisoned” (Table 1). In the regions surveyed, with few exceptions, “poisoned” refers to a syndrome consisting of abdominal swelling with accompanying constipation, swelling of extremities, acute and occasional body pains and aches; sometimes accompanied by anorexia/dysphagia. The underlying reason for the discomfort is thought to be sorcery or witchcraft directed at the affected individual. Overall, after “poisoning” the next most common (n = 23 [10.5%]) afflictions for which herbal treatments were recorded were pain, gynecological and gastrointestinal maladies. Treatments for pulmonary conditions and anemia or malnutrition ranked next (n = 21 [9.5%]), with malaria ranking number 8 (n = 20 [9.1%]) of the top overall categories of use. Unfortunately, these overall averages do not accurately represent the variation in the reported plant usages recorded in the different survey locations. While magic was the predominant condition treated overall, in the Marawaka area the largest category of medicinal plants use was for pulmonary diseases (17 of the overall total of 21 in that disease category). In the Bena dialect area, anemia and malnutrition had the highest number of plant uses recorded, 12 of the 37, with no species at this location recorded as being used to treat “poisoning”.

**Table 1:** Medicinally Utilized Plants in the Eastern Highlands of Papua New Guinea

**Genus and species [References]**	**Voucher number**	**Family**	**Local name [Dialect : Village]**	**Disease**	**Plant part**	**Medicinal preparation**	**Route**
*Abrus* sp. [9,11]	WL065	Leguminosae	Amanani-Aginogo [U : A]	GIT / Swelling [Magic]	L	Concoction	O
*Adenostemma* sp. [13,14]^*^, [15]^*^	SK057	Asteraceae	Hovepa [B : S]	Swollen skin & skin infection	L, F & vines	Sun drying	E
*Ageratum conyzoides* L. [8,9,11,13,14,16]^*^, [15]^*^	SK013	Asteraceae	Govu suva [S : O]	Diarrhea	L & F	F */* Succus	O
*Alpinia* sp. [8,9,11,13,14,16]^*^, [17]	WL052	Zingiberaceae	Amo-o [Ki : A]	GIT / Swelling [Magic]	Stem	Concoction	O
*Alpinia* sp. [8,9,11,13,14,16]^*^, [17]	SK056	Zingiberaceae	Sinogepuna [B : S]	Swollen joints	L	Chopped leaves are cooked together with pig meat	O
*Alpinia* sp. [8,9,11,13,14,16]^*^, [17]	SK030	Zingiberaceae	Gini giye [S : O]	Flatulence	yf L	Cut into pieces & cooked with pig meat in bamboo	O
*Alpinia* sp. [8,9,11,13,14,16]^*^, [17]	RJ006	Zingiberaceae	Koikadene [I : J]	Ovulation / Fertility	Fruit/Seeds	Fresh	O
*Alpinia* sp. [8,9,11,13,14,16]^*^, [17]	SK020	Zingiberaceae	Gavu [S : O]	Sever fever	yf L & shoot	Cut into pieces & cooked with pig meat in bamboo	O
*Alstonia brassii* Monach. [8-11,13,16,18]	SK064	Apocynaceae	Gigipe [B : S]	Skin infection	Sap (Milkywhite)	Fresh */* Succus	E
*Alstonia scholaris* (L.) R.Br. [8-11,13,16,18]	RJ011	Apocynaceae	Yaknalae's Ita [I : M-B]	Malaria	L, B	Fresh */* dry	O
*Alstonia* sp. [8-11,13,14,16]^*^, [15,18,19]^*^	SK046	Apocynaceae	Hihipa [B : S]	To gain strenght during warfare	L & B	Cut into small pieces & cooked with pig meat in bamboo	O
*Alyxia* sp. [14]^*^, [18]	RJ034a	Apocynaceae	Kevitingoje [I : J]	Dyspnea	soft L	Fresh	O
*Aristolochia indica* L. [20]	RJ001	Meliaceae	Yaki'Itchale [I : J]	Malaria, Abortion, uterine inflammation	L, B	Fresh, Decoction	O
*Ascarina philippinensis* C.B.Rob. [21]^*^, [14]^*^	RJ036	Chloranthaceae	Nebotniari [I : J]	High fever	L, fruit	Fresh	O
*Ascarina philippinensis* C.B.Rob. [21]^*^, [14]^*^	WL021	Chloranthaceae	Pawasa [F : Y]	GIT / Swelling [Magic]	L	Concoction	O
*Aspium* sp.	WL034	Unidentified	Waise [F : Y / E]	Diarrhea / abdominal pain / Cachexia	L	Concoction	O
*Bacopa* sp.	SK033	Scrophulariaceae	Momoto [G : U]	For gaining weight	L, F & Stem	Cut into pieces & cooked with pig meat in bamboo	O
*Barringtonia* sp. [8,9,13,15-17]^*^	SK054	Barringtoniaceae	Lahapa [B : S]	Stomach ache	L & sap	Sap & chopped leaves are mixed together and cooked with pig meat in bamboo	O
*Begonia rex* Putz.	RJ012	Begoniaceae	Woyatchale [I : J]	Sores / Boils (esp. on neck)	L, Stem	Fresh or dried	O
*Begonia* sp. [9,10,21]^*^, [13,14]^*^, [15,20,22-24]^*^	WL031	Begoniaceae	Awaya [F : Y]	Excessive menstrual bleeding	L	Concoction	O
*Begonia* sp. [9,10,21]^*^, [13,14]^*^, [15,22-24]^*^	RJ002	Begoniaceae	Saiguwong'giye [I : J]	Strong cough / general good health	L / or A	Fresh	O
*Bidens pilosa* L. [9,11,13,15,16,19,24]^*^	WL045	Asteraceae	Kamena [K : E]	Sores & Wounds	L	Heated */* Succus	E
*Bidens pilosa* L. [9,11,13,15,16,19,21,24]^*^	RJ013	Asteraceae	Toiporeyie [I : J]	Cuts/Wounds/Scratches (Hemostatis)	L & shoot	Succus (crushed)	E
*Blumea* sp. [9,10,13,14,21]	WL020	Asteraceae	Aleu [F : Y]	Weight decrease due to abdominal problems / nausea	L	Concoction	O
*Boerlagiodendron eminens* (W. Bull) Merr.***	SK050	Araliaceae	Sofa [B : S]	Control sexual desire of men	L	Leaves cut into pieces and cooked with pig meat in bamboo	O
*Bubbia* sp.	RJ021	Winteraceae	Pangwe [I : J]	Aches and pains, cough, arthritis, dyspnea	L, B	Decoction	O
*Calycacanthus magnusianus* K. Schum	RJ029	Acanthaceae	Ikowote/Wusale [I : W / AD]	Sores (all types)	L	Fresh (Mascerated)	E
*Castanopsis acuminatissima* (Blume) Rehder [10,21]^*^, [14]^*^	SK009	Fagaceae	Nege [S : G]	Otitis media	B	F */*Succus (Scraped and juice extracted)	E
*Casuarina oligodon* L.A.S. Johnson [21]^*^, [14]^*^, [25]	WL062	Casuarinaceae	Alaeva / Karavefa [Ki / K : A]	GIT / Swelling [Magic]	B	Concoction	O
*Chionanthus ramiflorus* Roxb.	SK045	Oleaceae	Hagavi [B : S]	Malnutrition/Growth retardation	L & B	Scrapped bark & chopped leaves are cooked in bamboo with pig meat	O
*Chrysanthemum* sp.	SK015	Asteraceae	Seveya [S : O]	Boil	F	F */*Succus	E
*Cinnamomum* sp. [9,10,21]^*^, [13,14]^*^, [15,20,22-24]^*^	WL007	Lauraceae	Yaravu [F : U]	Weakness / nausea / vomiting	B	Concoction	O
*Cinnamomum* sp. [9,10,21]^*^, [13,14]^*^, [15,20,22-24]^*^	SK036	Lauraceae	Yahoma [B : S]	Poison within body system	L & B	Scraped B is added to chopped L & pig meat and cooked in bamboo	O
*Cladonia scabriuscula* (Delise) Leight	WL075	Cladoniaceae	Lanefa-Kikinofa [K : A]	Vaginal discharge / bleeding	A	Heated	O
*Coleus blumei* Benth. [9,11]	SK043	Lamiaceae	Mufler [B : S]	Induce labour in childbirth	L	Cut into pieces and cooked with other leaves & pig meat in bamboo	O
*Coleus scutellarioides* (L.) Benth. [9,21]^*^, [13,15]^*^	RJ007	Lamiaceae	Wuririta [I : J]	Strong productive cough	f soft L	Heated and Fresh	O
*Commelina* sp. [8,9,11,14]^*^, [15]^*^	WL042	Commelinaceae	Hitirifa [K : E]	Headache [Analgesic]	A	Fresh	O
*Commersonia bartramiana* (L.) Merr. [8,21]^*^	WL001	Sterculiaceae	Kalaifa [K : E]	Emetic	B	Concoction	O
*Coprosma* sp. [14]^*^	WL066	Rubiaceae	Loanoya [Ki : A]	GIT / Swelling [Magic]	L	Concoction	O
*Cordyline fruticosa* (Linnaeus) A. Chevalier [8,13,14,16]^*^, [15]	SK019	Agavaceae	Gini fono [S : O]	Anemia & Dizziness	yL	Cut into pieces & cooked with pig meat in bamboo	O
*Cordyline terminalis* (L.) Kunth [9-11]	RJ044	Agavaceae	Alebiyei [I : KA]	Magical	soft un-sprouted shoot / L	Concoction	O
*Cosmos* sp.	SK001	Asteraceae	Seho gihi [G : N]	Toothache	F	Decoction	O (Rinse)
*Crassocephalum crepidioides* (Benth.) S. Moore [9,21]^*^, [14,15]^*^	RJ037	Asteraceae	Matchope [I : J]	Wounds and cuts (hemostasis)	L	Succus (crushed)	E
*Crassocephalum crepidioides* (Benth.) S. Moore [9,21]^*^, [14]^*^, [15]^*^	SK004	Asteraceae	Okore gihi [G : N]	Fresh cuts	yL, F & Stem	F */* Succus (All are squeezed together)	E
*Crassocephalum crepidioides* (Benth.) S. Moore [9,21]^*^, [14]^*^, [15]^*^	WL046	Asteraceae	Ino-Kamena-e [K : E]	Sores & Wounds	L	Heated */* Succus	E
*Crotalaria retusa* L. [9,13]	SK071	Leguminosae	Orupa flawa [G : G]	Chicken-pox	F	Fresh */* Succus	E
*Curculigo orchioides* Gaertn. [9]	SK005	Hypoxidaceae	Orupa napa [G: N]	Growth retarded children	Tubers	Maceration, then liquid is used to cook greens & meat in bamboo	O
*Cyrtandra* sp. [21]^*^, [13,14]^*^, [15]^*^	SK060	Gesneriaceae	Minise [B : S]	Malnutrition	L & Stem	Cut into pieces and cooked with pig meat in bamboo	O
*Cyrtandra* sp. [21]^*^, [13,14]^*^, [15]^*^	WL032	Gesneriaceae	Asaswa [F : Y]	Weight decrease due to abdominal problems / nausea	L	Concoction	O
*Cyrtandra* sp. [13,21]^*^, [14]^*^, [15]^*^	RJ015	Gesneriaceae	Kodatchale [I : J]	Emetic and strong cough (expectorant)	L	Fresh	O
*Decaspermum bracteatum* (Roxb.) A.J.Scott	SK047	Myrtaceae	Sosome [B : S]	Back & joint pain, headache & toothache	L & B	Cut into pieces and cooked with pig meat in bamboo	O
*Dendrobium* sp. [9,21]^*^, [13,14]^*^	SK029	Orchidaceae	Fonomuna [S : O]	Severe headache & dizziness	L & stem	Cut into pieces & cooked with pig meat	O
*Dendrobium* sp. [9,21]^*^, [13,14]^*^	WL048	Orchidaceae	Kinua [K : E]	Backache [Analgesic]	A	Fresh	O
*Dendrocnide interrupta* (L.) Chew	WL035	Urticaceae	Fai [K : U]	Muscle / joint pains / headache	L	Fresh	E
*Dendrocnide* sp. [13]	RJ048	Urticaceae	Iwole [I : J]	Productive cough	soft L	Concoction	O
*Desmodium* sp. [8,9,11,13,14,16]^*^, [22,26]	SK021	Leguminosae	Suwaroka ginona [S : O]	Malnutrition	L, F & Stem	Cooked with meat in bamboo	O
*Dianella ensifolia* (L.) DC. [14]^*^, [15]^*^	SK066	Liliaceae	Mamuha [B : S]	Irritating cough & throat mucus	L & Tubers	Infusion	O
*Diospyros* sp. [13]	WL010	Ebenaceae	Kigi Yosita-e [F : Y]	Shortness of breath w/ abdominal discomfort and bodyswelling	B	Concoction	O
*Diospyros* sp. [13]	WL037	Ebenaceae	HamaitoYosa [K : E]	Significant weight loss	L & B	Concoction	O
*Dodonaea viscosa Jacq*. [11,21]^*^, [14,16]^*^, [27]	WL044	Sapindaceae	Karu [K : E]	Bone Fractures / Painful joints	L	Heated	E
*Drymaria cordata* (L.) [9,14]^*^, [15]	RJ042	Caryophyllaceae	Motdeikedike [I : J]	Tooth ache	A	Decoction	O
*Elaeagnus* sp. [21]^*^	WL009	Elaeagnaceae	Tunakafe yosita-e [U : Y]	GIT / Swelling [Magic]	L	Dried	O
*Elaeocarpus* sp. [9,10,13,14,21]^*^, [15,17]^*^	WL051	Elaeocarpaceae	Ukari [K : A]	GIT / Swelling [Magic]	B	Concoction	O
*Eleutheranthera* sp. [28]	WL056	Asteraceae	Lemu [Ki : A]	Pains [general analgesic]	R	Concoction	O
*Emilia prenanthoidea* DC. [10,13,14]^*^, [9,21]	SK022	Asteraceae	Gambiri govu [S : O]	Rumbling stomach	L	Cut into pieces & cooked with pig meat in bamboo	O
*Erigeron sumatrensis* Retz*.*[14,21]^*^	WL013	Asteraceae	Kisekise / Okiopa [K : U]	GIT / Swelling [Magic]	B	Concoction	O
*Euphorbia hirta* L. [9,15,16]	SK032	Euphorbiaceae	Saha gihi [G : U]	Dysentery	L, F & Stem	F */* Succus	O
*Eurya* sp. [21]^*^, [14]^*^	WL070	Theaceae	Iyaleya [Ki : A]	Headache, fatigue, GI discomfort, muscle aches and arthralgia	L	Concoction	O
*Eustrephus latifolius* R. Br.	SK075	Philesiaceae	Haya sono [G : G]	Anti-helminthics	L	Fresh */* Succus	O
*Ficus benjamina* L. [29]	SK052	Moraceae	Makeyafa [B : S]	Joint & back pain	L & B	Scraped bark & chopped leaves are cooked with pig meat in bamboo	O
*Ficus copiosa Steud. *[8,9,21]^*^, [14,16]^*^	WL047	Moraceae	Atai-Yosita [K : E]	Chest Pains, labored breathing	L & B	Concoction	O
*Ficus pumila* L. [8-11,13,14,16]^*^, [19,22]	WL025	Moraceae	Kasakonde [F : Y]	Malaise, fever, shaking, vomiting [sometimes]	L	Concoction	O
*Ficus pungens* Reinw. ex BL. [9,10,13,14,16]^*^	SK073	Moraceae	Gegeha [G : U]	Shivering hands & feet	L & Fruit	Chopped leaves & smashed fruits are cooked together with pig meat in bamboo	O
*Ficus pungens* Reinw. ex Blume [9,10,13,14,16]^*^	RJ017	Moraceae	Ayeye [I : W]	Oral trush	soft f L	Succus (crushed)	O
*Ficus* sp. [8-11,13,14,16,21]^*^, [19,22]	WL015	Moraceae	Higifa [K : Y]	GIT / Swelling [Magic]	L	Concoction	O
*Ficus* sp. [8-11,13,14,16,21]^*^, [19,22]	WL038	Moraceae	Yagarueta [K : E]	Malaise, fever, shaking, vomiting [sometimes]	L	Concoction	O
*Finschia* sp.	SK037	Proteaceae	Yakof [B : S]	Abdominal swelling	L	Cut into pieces & cooked with pig meat	O
*Gardenia* sp. [14]^*^, [21]^*^	SK007	Rubiaceae	Amanini [G : N]	Faintness / Epilepsy	L	Chopped into pieces & cooked with meat in bamboo	O
*Glochidion* sp. [14]^*^, [15]^*^	SK024	Euphorbiaceae	Poroqui [S : O]	Frequent dizziness	yf L	Cut into pieces & cooked with pig meat in bamboo	O
*Gordonia papuana* Kobuski [21]^*^, [14]^*^	WL069	Theaceae	Oasiri / Kogaisiri [Ki / K : A]	GIT / Swelling [Magic]	B	Concoction	O
*Graptophyllum pictum* (L.) Griffith [8,21]^*^, [14]^*^, [22]	WL017	Acanthaceae	Otoro [U : Y]	Excessive menstrual bleeding	L	Concoction	O
*Grevillea papuana* Diels [10,13,21]	SK072	Proteaceae	Mumusopa yaha [G : G]	F cuts & Ulcer sores	L	Poultice	E
*Hemigraphis* sp. [8-10,21]^*^, [13,14]^*^, [20]	WL074	Acanthaceae	NamKatoga [K : NI (E)]	GIT / Swelling [Magic]	L	Concoction	O
*Hemigraphis* sp. [8-10,21]^*^, [13,14]^*^, [20]	RJ041	Acanthaceae	Bukmeye/Murie [I : KA]	Magical	L	Concoction	O
*Hyptis* sp. [9,11,13,23]	SK017	Labiatae	Seve moiba [S : O]	Spear wounds	L	F */* Succus	E
*Hyptis* sp. [9,11,13,23]	SK018	Labiatae	Gekesagu [B : S]	Otitis media	Soft stem & Stem skin	F */* Succus	E
*Impatiens hawkeri* W.Bull [10,21]^*^, [14]^*^	RJ043	Balsaminaceae	Meyebui [I : KA]	Magical	L	Concoction	O
*Impatiens hawkeri* W.Bull [10,21]^*^, [14]^*^	RJ045	Balsaminaceae	Kolumbota [I : KA]	Magical	L	Concoction	O
*Kibara katikii* Philipson	SK025	Monimiaceae	Suwa nara [S : O]	Epilepsy	L	Cut into pieces & cooked with pig meat in bamboo	O
*Laportea decumana* (Roxb.) Wedd. [9,10,21]^*^, [13,16,19,22,24,26,30]	RJ009	Urticaceae	Nabotne (green) [I : J]	Liver pain	soft L	Heated and Fresh	O
*Laportea decumana* (Roxb.) Wedd. [9,10,21]^*^, [13,16,19,22,24,26,30]	RJ018	Urticaceae	Nabotne (Pink) [I : J]	Joint pain (analgesic); magical	L	Fresh	O
*Laportea* sp. [9,10,21]^*^, [13,14,16,19,22,24,26,30]	RJ004	Urticaceae	Keletichale [I : J]	Liver pain (Epigatric pain)	f soft L	Fresh	O
*Leucosyke capitellata* (Poir.) Wedd. [8]	SK063	Urticaceae	Hagaza [B : S]	Skin itchiness	L & stem	Fresh */* Succus	O & E
*Litsea* ex*sudens* Kosterm. [14]^*^	SK048	Lauraceae	Sagifa [B : S]	Cleanes bodysystem	L & B	Leaves & barks are cut into pieces and cooked with pig meat in bamboo	O
*Ludwigia hyssopifolia* (G. Don) exell apud A.R. Fernandes [21]^*^	WL040	Onagraceae	Lawolawosa [K : E]	Blood in urine, yellow tongue, headache with high fever, aching joints [all ocurring together]	A	Concoction	O
*Ludwigia octovalvis* (Jacq.) Raven [14]^*^	SK069	Onagraceae	Goluwaiyo [G : G]	Ulcer sores	Fruit & seeds	Fresh */* Succus	E
*Maclura* sp.	RJ020	Moraceae	Iveriate [I : J]	Malaria	soft L	Fresh	O
*Medinilla* sp.	SK028	Melastomataceae	Lunu aira [S : O]	Anemia	L	Cut into pieces & cooked with pig meat	O
*Melastoma malabathricum L. *[14]^*^	WL014	Melastomataceae	Tawakaya [F : U]	GIT / Swelling [Magic]	L & B	Concoction	O
*Melastoma* sp. [8,9,11,13,14]^*^	WL006	Melastomataceae	Kora Yosa / Au Yosaita [F / K : Y]	Heart Problems	L	Concoction	O
*Mucuna stanleyi* C.T.White [8,9,13]	RJ014	Leguminosae	Kang'ole [I : J]	Contraception	soft L	Succus (crushed)	O
*Murraya paniculata* (L.) Jack [28]	SK068	Rutaceae	Onupa yaha [G : N]	Mentallydisturbed	L	Leaves are cut into pieces and cooked with meat in bamboo	O
*Murraya* sp. [28]	SK031	Rutaceae	Nuva yaha [G : G]	Nasal congestion	L	Steaming	I
*Musa* sp. [8,9,11,13,14,16,21]^*^, [23,24,30]	WL053	Musaceae	Abu [Ki : K]	GIT / Swelling [Magic]	Stem	Concoction	O
*Myrmecodia* sp. [8,21]^*^, [13]	RJ046	Rubiaceae	Kleklina [I : KA]	Magical	soft L	Concoction */* Fresh	O
*Ophiorrhiza nervosa* RidL. [14]^*^	SK049	Rubiaceae	Atu [B : S]	Poison within body system	L	Cooked with pig meat in bamboo	O
*Ophiorrhiza* sp. [14,21]^*^	WL011	Rubiaceae	Temu / Afela Hefi [F / K : Y]	Epigastric pain, nausea, vometing, abdominal swelling [Stomach cancer]	L & soft stem	Concoction	O
*Orania* sp.	WL072	Arecaceae	Vayave [K : AN (E)]	Difficult breathing, cough and wheezing	B	Concoction	O
*Oxalis corniculata* L. [9,14,16]^*^, [19]	SK041	Oxalidaceae	Sokolo [B : S]	Dysmenorrhoea	L, F & Stem	Cut into pieces & cooked with pig meat in bamboo	O
*Oxalis corniculata* L. [9,14,16]^*^, [19]	SK035	Oxalidaceae	Gehani gihi [G : M]	Labour pain	L, F & Stem	F */* Succus	O
*Pandanus* sp. [9,13,14,16,21]^*^, [22]	RJ047	Pandanaceae	Awangwe [I : J]	Asthma	soft L	Succus (squeezed)	O
*Phyllanthus niruri* L. [9,11,13,16]	SK070	Euphorbiaceae	Saha gihi [G : G]	Stings from insects/bees	L & F	Fresh */* Succus	E
*Piper aduncum* L. [29]	WL043	Piperaceae	Kamani-Lucefa [K : E]	Bone Fractures	L	Heated	E
*Piper betle* L. [9,10,16]	SK061	Piperaceae	Yagerere [B : S]	Malnutrition	L	Chopped and cooked together with pig meat	O
*Piper gibbilimbum* C.DC. [14]^*^, [31,32]	RJ038	Piperaceae	Kutmunne [I : J]	Improves platability of other TrM	L	Wrapper	O
*Piper* sp. [8-10,21]^*^, [13,14,16]^*^, [17,18,22]	RJ039	Piperaceae	Mudutganan'ne [I : KA]	Magical	soft L	Concoction	O
*Piper* sp. [8-10,21]^*^, [13,14,16]^*^, [17,18,22]	SK026	Piperaceae	Gegiya [G : U]	Toothache	yf L	Succus	E
*Pipturus argenteus* (G.Forst.) Wedd. [9,10,13,16,18,26]	SK067	Urticaceae	Ase yaha [G : N]	Remove worms	L	Chopped leaves are cooked together with pig meat	O
*Pittosporum sinuatum* BL. [14]^*^	WL058	Pittosporaceae	Ilawau-u [Ki : A]	GIT / Swelling [Magic]	L	Concoction	O
*Pittosporum* sp. [9,21]^*^[13,14,16]^*^	RJ027	Pittosporaceae	Wununghe [I : W	Scabies, ulcerative sores	A	Decoction	O
*Planchonella* sp. [14]^*^	RJ024	Sapotaceae	Ik'klake [I : J]	Abdominal swelling / Magical	Seed, L, B	Decoction (bark) */* fresh	O
*Plantago major* L. [21]^*^	SK044	Plantaginaceae	Getuya [B : S]	Diarrhea	Whole	Either cooked separately or with pig meat in a bamboo	O
*Plantago major* L. [21]^*^	SK034	Plantaginaceae	Masiga gihi [G : U]	Deep wound (pig/dog bite, axe/knife wound)	L & stem	F */* Succus	E
*Plectranthus scutellarioides* R. Br. [3,13,14,16,18]	SK040	Lamiaceae	Salita [B : S]	Dysentery	L	Cut into pieces & cooked with pig meat in bamboo	O
*Podocarpus neriifolius* Don. [9,27]	WL054	Podocarpaceae	Laso [Ki / K : A]	GIT / Swelling [Magic]	B	Concoction	O
*Poikilospermum* sp. [8,14]^*^	SK058	Urticaceae	Sikrutafa [B : S]	To gain weight/become fat	L & vine	Chopped leaves & scraped vines are cooked together with pig meat	O
*Polygala paniculata* L., [21]^*^, [13], [14]^*^	SK016	Polygalaceae	Nuva gihi [G : O]	Malaria & High Fever	L & F	Cooked with meat in bamboo	O
*Polygonum strigosum* R. Br. [14]	RJ022	Polygonaceae	Tungole [I : J]	Labor pain (analgesic)	soft L	Fresh	O
*Polyscias filicifolia* (C.Moore ex E.Fourn.) L.H.Bailey	RJ005	Araliaceae	Iriduki'Imetchale [I : J]	Malaria	L	Fresh	O
*Psidium guajava* L. [8,9,13,16]	SK002	Myrtaceae	Gusigusi [G : N]	Diarrhea	L	Fresh */* Succus	O
*Psoralea* sp.	SK003	Leguminosae	Gulumeha [G : G]	White mouth sores / Candida like	L, F & Stem	F */* Succus (All at once)	O (Chewed)
*Psychotria* sp. [9,11,21]^*^, [13,14]^*^	WL033	Rubiaceae	Katoya [F : Y]	GIT / Swelling [Magic]	L	Concoction	O
*Rubus moluccanus* L. [9,14,16,21]^*^	WL023	Rosaceae	Uruturu [F : Y]	Fevers	L	Concoction	O
*Rungia klossii* S.Moore [11,21]^*^, [14,30]^*^	WL004	Acanthaceae	Hefi [K : U]	none	Herb	Major additive to Wl001- > 003	O
*Saccharum* sp. [9,11,21]^*^, [13,14,16]^*^, [17,23,26]	RJ051	Poaceae	Sale / Saimije [I : J]	Improves platability of other TrM	Crystallized	Additive (taste */* texture)	O
*Sacciolepis* sp. [10,13,14,21]	WL036	Poaceae	Hufa-aigoyae [K : AN]	Small sores & minor cuts	F / Stem	Succus	E
*Salacia* sp. [14]^*^	WL029	Celastraceae	Agenafa [K : Y]	GIT / Swelling [Magic]	L & Stem	Concoction	O
*Sambucus javanica* Reinw. ex Blume	SK008	Caprifoliaceae	Golani yaha [G : N]	Chicken-pox	F	Infusion used for bathing	E
*Sanchezia nobilis var. glaucophylla* Lem.	SK076	Acanthaceae	Girukaru [G : U]	Broken bones & joint dislocations	L	Cooked with meat or other greens	O & E
*Saurauia andreana* (F.MuelL.) Diels [8,14]^*^	WL064	Actinidiaceae	Ayano [Ki : K]	GIT / Swelling [Magic]	L	Concoction	O
*Saurauia* sp. [8,21]^*^, [14]^*^	SK027	Actinidiaceae	Goiva [S : O]	Ulcers	L	Poultice	E
*Saurauia* sp. [8,21]^*^, [14]^*^	SK051	Actinidiaceae	Kiahuyave [B : S]	Dysmenorrhoea	L & B	Leaves */* bark or both are cut into pieces and cooked with pig meat in bamboo	O
*Saurauia* sp. [8,21]^*^, [14]^*^	SK053	Actinidiaceae	Yaketupa [B : S]	Abnormal mouth & nose bleeding	L & B	Chopped leaves & scraped bark are cooked together with pig meat in bamboo	O
*Saurauia* sp. [8,21]^*^, [14]^*^	RJ010	Actinidiaceae	Kokgingila [I : J]	Permanent contraceptive	B	Decoction	O
*Saurauia* sp. [8,21]^*^, [14]^*^	RJ030	Actinidiaceae	Gol'lik [I : W / AD]	Liver pain / epigastric pain	L	Fresh	O
*Saurauia* sp. [8,21]^*^, [14]^*^	WL005	Actinidiaceae	Karakieta [F : Y]	Pains, Fevers & GI Symptoms ["typhoid symptoms"]	L	Concoction	O
*Saurauia* sp. [8,21]^*^, [14]^*^	WL018	Actinidiaceae	Kaware [F : Y]	Excessive menstrual bleeding	L	Concoction	O
*Schefflera* sp. [21]^*^, [13], [14]^*^, [15]^*^	WL003	Araliaceae	Ase [K : E]	Emetic	L	Concoction	O
*Scindapsus* sp.	SK055	Araceae	Yage [B : S]	Severelysick patients	Stem & yf L	Chopped leaves & scraped vines are cooked together with pig meat	O
*Selaginella emmeliana* Van Geert	WL049	Selaginellaceae	Kakasi [Ki / K : A]	Swollen Hands and Feet	L	Concoction	O / E
*Senecio* sp. [14]^*^	SK012	Asteraceae	Gotu aira [S : O]	Cough with runny nose	yL & S	F */* Succus	O
*Setaria* sp. [8-10,14]	SK010	Poaceae	Nomu suva [S : O]	Malaria	whole plant	Chopped into pieces & cooked with meat in bamboo	O
*Sida acuta Burm.* F. [9,13,16]	SK014	Malvaceae	Nivinihani [G : O]	Dysmenorrhoea, Diarrhea	yf L & Rs	Eaten with food & F */* Succus	O
*Sigesbeckia orientalis* L. [14]^*^	SK042	Asteraceae	Mezamua [B : S]	Diarrhea	Whole	Fresh */* Succus	O
*Smilax rotundifolia* L. [8,9,16]	WL024	Smilacaceae	Kogana [K : Y]	Pains and Fevers [Analgesic]	L	Concoction	O
*Smilax* sp. [8,9,14,16]^*^	WL030	Smilacaceae	Ala-Kogana [K : U]	GIT / Swelling [Magic]	L	Concoction	O
*Smilax* sp. [8,9,14,16]^*^	SK023	Smilacaceae	Gatapa nara [S : O]	Epilepsy	y soft L	Cut into pieces & cooked with pig meat in bamboo	O
*Steganthera* sp. [14]^*^	RJ033	Monimiaceae	Kwagisal'lik [I : AD]	Strong cough with burning/nausea	soft L & S	Fresh	O
*Symplocos* sp. [14,21]^*^	WL002	Symplocaceae	Vefa [K : E]	Emetic	B	Concoction	O
*Symplocos* sp. [14,21]^*^	WL059	Symplocaceae	Nimihavaya [Ki : A]	Epigastric pain	L	Concoction	O
*Symplocos* sp. [14,21]^*^	RJ032	Symplocaceae	Guruke [I : W	Cough	L	Fresh	O
*Syzygium longipes* Merr. & L.M.Perry [8-11,13,16,18]	SK059	Myrtaceae	Maze [B : S]	Malnutrition	L & B	Cut into pieces and cooked with pig meat in bamboo	O
*Syzygium* sp. [8-11,21]^*^, [13,14,16]^*^, [18]	SK074	Myrtaceae	Nuva yaha [G : G]	Mild-severe chest pain	L & Fruit	Dry */* fresh chopped leaves are cooked with meat in bamboo	O
*Syzygium* sp. [8-11,21]^*^, [13,14,16]^*^, [18]	WL019	Myrtaceae	Yasawa [F : Y]	GIT / Swelling [Magic]	B	Concoction	O
*Syzygium* sp. [8], [9-11,21]^*^, [13,14,16]^*^, [18]	SK038	Myrtaceae	Fitome [B : S]	Swallowing of poison	L & B	Cut into pieces and cooked with pig meat in bamboo	O
*Tagetes* sp. [11]	SK006	Asteraceae	Ukuromini [G : N]	Toothache	F & Stem near F	F (All are squeezed and applied directly)	D
*Tasmannia piperita* (Hook. f.) Miers. [13,14]	RJ008	Winteraceae	Iridukichale [I : J]	Malaria and strong cough	L	Fresh or dry	O
*Tecoma* sp. [21]^*^, [15]^*^	WL067	Bignoniaceae	Na-a [Ki : A]	Vaginal discharge/bleeding	R	Concoction	O
*Timonius belensis* Merr. & L.M.Perry [14]^*^	SK039	Rubiaceae	Kipe [B : S]	Joint & back pain	L & B	Scraped B is added to chopped L & pig meat and cooked in bamboo	O
*Timonius* sp. [9,13,14,16]^*^	WL039	Rubiaceae	Ubagu [K : E]	GIT / Swelling [Magic]	L	Concoction	O
*Trichospermum* sp.	WL071	Tiliaceae	Aviya [Ki : A]	Headache, fatigue, GI discomfort, muscle aches and arthralgia	L	Concoction	O
*Tridax procumbens* L. [11,16]	SK077	Asteraceae	Lilita [G : U]	Mild-severe headache	L	Cooked with meat in bamboo	O
*Tristiropsis* sp.	RJ023	Sapindaceae	Longola [I : J]	Malaria, cough w/ chest pain / Magical	soft L	Fresh	O
*Uncaria* sp. [8,21]^*^, [14]^*^	SK062	Rubiaceae	Glagigo [B : S]	Internal sores & candida infections	L & Stem	Candida: stem smashed to release succus which is gargled; sores: stem and leaves are cooked and eaten	O (Rinse) / O
Unidentified	SK011	Araliaceae	Gororotave nara [S : O]	Toothache	L & stem	F */* Succus (Cut & squeezed in hand)	E
Unidentified	SK065	Not given	Nagepa [B : S]	Headache	L, F & Stem	All parts are cooked together with pig meat	O
Unidentified	RJ003	Unidentified	Imetchale [I : J]	Malaria	L	Fresh	O
Unidentified	RJ016	Icacinaceae	Kawikaule [I : J]	Strong cough	L	Fresh or dry	O
Unidentified	RJ025	Scrophulariaceae	Imetchale [I : J]	Malaria and Asthma	A	Fresh or dry	O
Unidentified	RJ026	Solanaceae	Kwagi'Kuwogie [I : J]	Persistent cough	L	Fresh	O
Unidentified	RJ028	Unidentified	Kimamite (small) [I : J]	Malaria / mental enhancement	L, Stem	Leaf Fresh */* Stem charred and inhaled	O
Unidentified	RJ031	Unidentified	Kat'tungje [I : J]	Strong cough with rhinorrhea (decongestant)	L	Fresh	O
Unidentified	RJ034b	Unidentified	Ammate [I : J]	Dyspnea	soft L	Fresh	O
Unidentified	RJ035	Unidentified	Kimamite (big) [I : J]	Headache / Malaise	L	Fresh	O
Unidentified	RJ040	Euphorbiaceae	Klalebongbonge [I : KA]	Magical	soft shoot / L	Concoction	O
Unidentified	WL008	Leguminosae	Orovia [F : Y]	Abdominal discomfort / bodyaches [also magical: make people like bearer]	F	Fresh	O
Unidentified	WL012	Unidentified	Kuougo [U : Y]	GIT / Swelling [Magic]	Stem [dried]	Concoction	O
Unidentified	WL016	Euphorbiaceae	Kamira [KT : F]	Excessive menstrual bleeding	L	Concoction	O]
Unidentified	WL026	Unidentified	Tewesa [F : Y]	GIT / Swelling [Magic]	A	Concoction	O
Unidentified	WL027	Myrtaceae	Kuta [K : Y]	GIT / Swelling [Magic] [also magical as protection for bearer]	B	Concoction	O
Unidentified	WL028	Unknown	Nonhe [F : Y]	GIT / Swelling [Magic]	L & B	Concoction	O
Unidentified	WL041	Unidentified	Eso-Aginogi [K : E]	Weight Loss with severe bodyaches and GI complications	A	Concoction	O
Unidentified	WL050	Unidentified	Veya [Ki : A]	Swollen Hands and Feet	A	Concoction	O / E
Unidentified	WL055	Lamiaceae	Natano [Ki / K : A]	GIT / Swelling [Magic]	B	Concoction	O
Unidentified	WL057	Sapindaceae	M-e [Ki : A]	Pains [BodyAches]	B	Concoction	O
Unidentified	WL060	Theaceae	Alau / Kalau [Ki / K : A]	GIT / Swelling [Magic]	B	Concoction	O
Unidentified	WL061	Unidentified	Kaviaya / Kamagafa [Ki / K : A]	GIT / Swelling [Magic]	B	Concoction	O
Unidentified	WL068	Melastomataceae	Masirita [Ki : A]	GIT / Swelling [Magic]	L	Concoction	O
Unidentified	WL076	Unidentified	Hapalaga Hitirifae [K : NI (E)]	Diarrhea	F / Fruit	Fresh	O
Unidentified	RJ050	Unidentified	Tungoje [I : J]	Cough	L	Fresh	O
*Usnea strigosa* (Ach.) Eaton	WL063	Parmeliaceae	Oleazu [Ki : A]	Headache	A	Concoction	O
*Vaccinium* sp. [13], [14]^*^, [21]^*^, [15]^*^	RJ019	Ericaceae	Yopengchale [I : J]	Strong cough, asthma/dyspnea, fertility, stop menstruation	L	Fresh	O
*Zingiber officinale* Roscoe [8-10,16,22,26,30]	RJ059	Zingiberaceae	Imate [I : J]	Sore throat / Magical	Rhizome	Concoction	O
*Zingiber officinale* Roscoe [8-10,16,22,26,30]	WL073	Zingiberaceae	Atu [K : NI (E)]	Common Ingredient	R	Concoction	Many

**Languages**: Gahuku – G; Siane – S; Bena – B ; Ipma-Baruya Language – I ;Fore – F ; Keyagana – K ;Kimi = Ki ; Unknown – U.

**Villages**: Amusa - A; Ande - AD; Aniruvi - AN; Egera-Haga - E; Ginipauka - G; Jomuru -J; Kimi - K; Kam'mwa - KA; Kamiyovindi-Tunuku - KT; Masiga - M; Mala and Boiko M-B; Numurapoka - N; Niruvi - NI; Orumba - O; Sekagu -S; Unknown - U; Wauko - W; Yawanita Haya -Y.

**Route**: Oral – O ; External – E; D – direct on tooth; I - Inhaled.

**Plant parts**: Aerial parts – A; Bark – B; Flowers – F;; Leaves – L; Tubers – T; Root – R; Vine – V; Whole – W; young – y.

**Medicinal preparation**: Fresh – F.

*** now known as *Osmoxylon eminens* (W.Bull) Philipson.

A * following a reference number indicates no reference to medicinal use of the plant was made in the cited source.

### Plant parts utilized, preparation and administration

**Figure 2:**
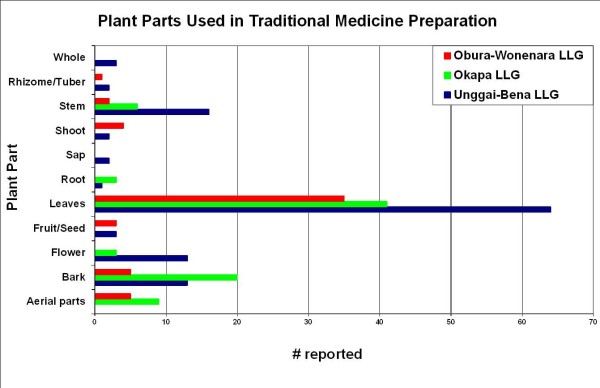
The plant parts used for traditional medicines in the Eastern Highlands by study area.

**Figure 3:**
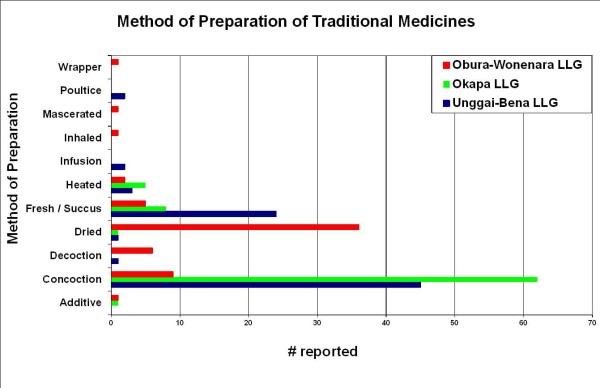
**Methods of preparation of medicinal plants prevalent in the Eastern Highlands region of PNG.** (The use of a plant as a wrapper/container to prepare medicines may not imply medicinal action by itself).

**Figure 4:**
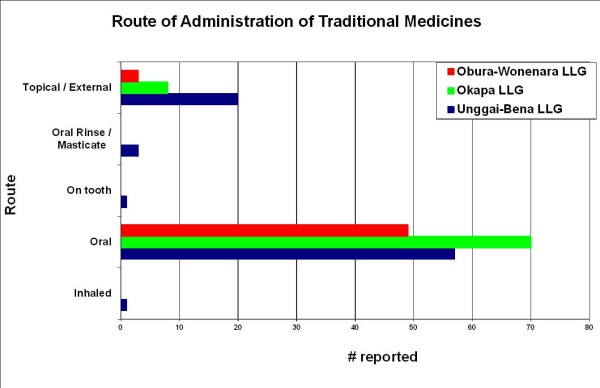
**Route of consumption of medicinal plants and preparations by frequency of use or mode.** Note: For reasons of clarity the language groups have been folded into LLGs (Unggai-Bena LLG for Bena, Gahuku and Siane languages; Okapa LLG for Fore, Kimi and Keyagana languages and Obura-Wonenara LLG for Ipma-Baruya language).

Overall, leaves were most commonly used in the preparations of the healers interviewed (Figure 2). This was followed by bark and stems as the next most likely plant parts to be used. Exceptions to this rule were the reports from Gahuku and Siane language groups where stems and flowers were reported to be used more frequently than bark. The mixture of two or more ingredients is the most common mode of preparation recorded in the Eastern Highlands, except for the Gahuku and Marawaka regions where the consumption of fresh material or fresh succus (expressed juice) was reported as more common (Figure 3). Also reported was the occasional practice of the storage and consumption of dried medicinal plants. Throughout the Eastern Highlands oral consumption of medicinal plants was the most common administration practice recorded (Figure 4). Topical application was the second most widely practiced mode of administration.

### Commonly used plant species among healers that were interviewed

*Ascarina philippinensis* is a fairly widespread tree in the PNG highlands [33]. Reports of its medicinal use are uncommon, although its use in ritual food preparation (“kirai” in the Chimbu region of the highlands) for which health promotion is a component of the ceremony, has been reported [21]. A concoction incorporating leaves of *Ascarina philippinensis* is used by the Fore to treat "poison", however in Marawaka the fresh leaves and fruit are used to treat high fevers and weight loss. In both areas the preparations are consumed with other ingredients, in the Fore area after heating.

*Bidens pilosa* is a medicinal plant used widely in PNG, often applied topically for sores and boils. The freshly squeezed succus from leaves of *Bidens pilosa* is utilized by the Keyagana group to treat sores and wounds, in the Marawaka area the succus of leaves and shoots is used for the same conditions. In both areas the application method is topical. *B. pilosa* is a common weed widely distributed from low altitudes to over 2,000 m in Papua New Guinea [16]. An additional recorded use is the use of fresh leaves, gently heated, and placed over the affected eye to treat red or sore eyes [9,16].

*Crassocephalum crepidioides*, is widespread in subtropical and tropical areas around the World. Succus from fresh leaves is used in the Gahaku areas for fresh cuts and wounds, in the Kayagana group the leaves are heated and then succus is used for sores and wounds. In both cases application is topical. Leaves and petioles have been reported to be heated and crushed and applied to sores in the Mt. Hagen area of the highlands [13], and the application of crushed leaves to sores was reported by Holdsworth from Goodenough Island [9]. *Crassocephalum crepidioides* is widespread in subtropical and tropical areas around the world. Antimalarial and antimicrobial dihydroisocoumarin compounds have been isolated from an endophytic *Geotrichum sp.* fungus of this plant [34], although there is no evidence that the coumarin compounds contribute to the putative medicinal properties of this plant.

Gahaku-speaking people cook chopped leaves and fruit of *Ficus pungens* and use them to treat "shivering hands and feet", while in Marawaka liquid extract from fresh leaves is used to treat thrush (oral candidaiasis). In both cases the traditional medicines are consumed orally. *Ficus pungens* is common throughout the lowlands of Papua New Guinea [33] and there are many reports of its medicinal use in PNG [9,10,13,14,16]. Its use for coughs has been repeatedly reported; e.g., in Holdsworth [9] coughs are treated in the Sepik by swallowing sap obtained from the root. Also, the leaves of *Ficus pungens* are crushed together with the leaves of a species of *Mallotus*, and mixed with water and consumed to relieve a bad cough in Buka, Bougainville [9]. A solution made from crushed and squeezed bark is taken orally for a week for treatment of asthma [16]. Other reported uses include: in the previous Northern District of PNG, leaves were heated over a fire and applied topically to alleviate body pains [13,35]. Also, the Traditional Medicine Database records the use of fresh leaves in the treatment of inguinal hernia (swelling of testicles) by brushing the leaves upwards against the testicles [16]. The application of leaves to sores was also reported by Telban [10].

Both *Oxalis corniculata* and *Plantago major* are used in Bena and Gahaku speaking communities. In Bena areas *Oxalis corniculata* is cooked with pig meat and eaten to treat painful menses, while in Gahaku areas its fresh fruit and flowers, as well as freshly squeezed succus, are recorded as consumed orally to relieve labor pains. *Oxalis corniculata* is found everywhere in Papua New Guinea, but most commonly in the Highlands [33]. Traditional uses include pulping of the whole plant to extract sap that is drunk to treat syphilis and prostate cancer [16]. To treat burns, the whole plant is crushed, chewed and spat onto the burn [9].

According to Zubair [36], “*Plantago major* (common plantain) has been used in folk medicine all over the world, mainly for the healing of wounds.” We have found reports of its medicinal use in PNG are uncommon, however. *Plantago major* is cooked whole in Bena areas and consumed orally as treatment for diarrhea, while in Gahaku areas leaves and succus from stems are reported to be applied topically to treat deep wounds.

*Zingiber officinale* is found in many preparations in Kaygana speaking areas where the rhizome is used as an additive to improve taste and texture of medicinal preparations; however in Marawaka it is used in a concoction to treat sore throat and for magical purposes. *Zingiber* is grown as a culinary and ornamental plant [16]. It is widely used for a plethora of indications too numerous to list here e.g., [8-10,16,21,22,26,30].

### Lesser known medicinal plant species of PNG

It is frequently the case in reports such as this that it is not always possible to find references for medicinal use in PNG for all the species listed. Plants identified to the species level reported here, but not found listed as medicinal in the PNG literature we reviewed, include: *Begonia rex*, which is consumed orally for boils and sores, including tropical ulcers, in the Ipma-Baruya language area. There are several *Begonia* species reported as used medicinally in PNG [9,10,13,14,20,22-24], but not *B. rex*. So far as we can tell the species *Begonia rex* has only been reported from West Papua to this point [33]. Likewise, there are records of *Calycacanthus magnusianus* in PNG [33], but not of its medicinal use. This is also the case for other plants listed in this report: *Ascarina philippinensis*; *Chionanthus ramiflorus*; the lichen *Cladonia scabriuscula*; *Decaspermum bracteatum*; *Dianella ensifolia*; *Eustrephus latifolius*; *Gordonia papuana*; *Kibara katikii*; *Litsea exsudens*; *Ludwigia hyssopifolia*; *Ludwigia octovalvis*; *Melastoma malabathricum*; *Ophiorrhiza nervosa*; *Pittosporum sinuatum*; Selaginella emmeliana*, a spike moss; *Sigesbeckia* orientalis; *Timonius belensis* and Usnea strigosa*; (* Known to exist in PNG, personal communication, Prof. Robert Johns, Botanical Research Institute of Texas, New Guinea Research Program).

Leaves of *Boerlagiodendron eminens*, also known as *Osmoxylon eminens*, were recorded in Sekagu village, Bena speaking area, to be cut and eaten to suppress sexual desire in men. This plant is not listed in the PNG Plants database [33] and maybe a unique record. Blackwood [22] reported leaves from a *Boerlagiodendron* sp. to be eaten by the Kukukuku people of the Highlands. Other lesser known medicinal plants in this report that are not represented in PNG Plants database [33] include *Dendrocnide interrupta* (*Fleurya interrupta*); *Polyscias filicifolia*; *Sambucus javanica*; and *Sanchezia nobilis* var. glaucophylla. We assume that *Sanchezia* is likely recently introduced as an ornamental species.

## Conclusions

As described by Feil [37], “Highlanders may inhabit similar altitudes but within this range there are distinct sub regions…” The Eastern Highlands are relatively drier and historically became heavily populated later than the wetter western regions. The contemporary cultural and botanical diversity of the Eastern Highlands, the continued reliance on medicinal plants in rural communities there, and the innovation and experimentation of the practitioners, combine to yield a rich and dynamic area for study. While many traditional practitioners continue to treat symptoms attributed to “witchcraft”, modern perceptions of disease etiology are penetrating even remote villages, resulting in mixed rationales for giving treatments that reflect these changes in understanding.

Several of the species reported here are used in the Eastern Highlands but not frequently reported in the literature for medicinal use elsewhere in PNG. These include both plants that are known to be widely distributed in PNG (e.g., *Ascarina philippinensis* and *Calycacanthus magnusianus*) and also those that are not known to be widely distributed in PNG (e.g., *Boerlagiodendron eminens* and *Aristolochia indica*). The medicinal use of those plants that are localized geographically might reflect potent activity accessible only to those fortunate enough to live there, while the unique use of widely distributed plants might reflect either the local discovery of a useful property, or experimentation of local healers in plant use – practices perhaps not yet spread to or reproduced elsewhere in PNG. In any case, efforts to validate the utility of PNG medicinal plants cannot completely skirt these issues. Subjecting traditional plant preparations to laboratory testing can demonstrate specific bioactivities that may help validate the traditional use, but unfortunately, it is beyond the capacity of the PNG Department of Health or the University system to test for all the bioactivities represented in the wide range of plant uses recorded by the surveys. In these circumstances, the consistent use of a particular plant for specific indication by a large number of healers, across a wide range of ethnic traditions, is notable and may support the notion of a particular herbal remedy’s efficacy.

The effort to document and validate medicinal plant use in PNG is part of a larger strategy that is under way to meet the health care needs of citizens of PNG. As described by Waruruai et al. [8], the Traditional Medicines survey project complements other programs supported by the PNG Ministry of Health to promote the use of efficacious herbal remedies in underserved communities. Traditional healer associations have already been established in several provinces and basic manuals on diagnosis and plant use have been drafted. The aim is to promote integrated medical treatment options in an approach to health care that combines effective and accessible traditional practices with Western protocols (when available). The medicinal plant surveys reported here are the product of collaboration amongst the faculty at UPNG and the University of Utah, with support provided by the Fogarty International Center of the NIH, USA [38], and the PNG Ministry of Health. The Traditional Medicines survey project utilizes a university training tutorial as one component of a larger strategy aimed at providing improved health care options to a burgeoning population.

## Competing interests

The authors declare that they have no competing interests.

## Authors’ contributions

PR and TM with colleagues established the Traditional Medicines survey program at UPNG and oversaw training and support of students and the maintenance of the Traditional Medicines Database. RJ, SK and WL, senior year Bachelor of Pharmacy students travelled to their respective Eastern Highlands communities and conducted the interviews with locally acknowledged healers and performed the corresponding plant collections and documentation. PP and OG oversee the UPNG herbarium and identified collected plants. MK and LB integrated the data from the three surveys, drafted the manuscript and performed the literature analysis. All authors read and approved the final manuscript.

## Supplementary Material

Additional file 1 Information sheet on traditional herbal preparations and medicinal plants of Papua New Guinea. Click here for file
