# Supplementary material for: An ethnobotanical survey of medicinal plants used in the eastern highlands of Papua New Guinea
Source: J Ethnobiol Ethnomed. 2012 Dec 18;8:47. doi: 10.1186/1746-4269-8-47 (PMC3549922; doi:10.1186/1746-4269-8-47)
Supplement: Additional file 1 — Information sheet on traditional herbal preparations and medicinal plants of Papua New Guinea. [file 1746-4269-8-47-S1.pdf]

DISCIPLINE OF PHARMACY
SCHOOL OF MEDICINE & HEALTH SCIENCES
UNIVERSITY OF PAPUA NEW GUINEA

INFORMATION SHEET ON TRADITIONAL HERBAL PREPARATIONS AND MEDICINAL PLANTS OF PAPUA NEW GUINEA

(One form should be completed for each plant or preparation)

Date: ………………

1. Family name of plant: …………………………………………………………..

2. Genus & species: ……………………………………………………………….

3. Common English name: ………………………………………………………

4. Local name(s) (specify which dialect/language):
……………………………………………………………………………………….
……………………………………………………………………………………….
……………………………………………………………………………………….
……………………………………………………………………………………….
5. Herbarium specimen number and address of the herbarium where a specimen of this plant has been deposited:
………………………………………………………………………………………..
………………………………………………………………………………………..
………………………………………………………………………………………..
6. Location of plant (i.e. which part of town/village/LLG): …………………………
……………………………………………………………………………………….
……………………………………………………………………………………….
……………………………………………………………………………………….

7. Diseases, symptoms and conditions for which plant is used:
………………………………………………………………………………………
………………………………………………………………………………………
………………………………………………………………………………………
………………………………………………………………………………………
………………………………………………………………………………………
(Be as specific as possible)

8. Plant parts used: ……………………………………………………………….

9. Method (s) of preparation for use (Provide details about amount of plant part required, whether fresh or dried, ratio of plant to solvent, etc.):
……………………………………………………………………………………..
……………………………………………………………………………………..
……………………………………………………………………………………..
……………………………………………………………………………………..
……………………………………………………………………………………..

…………………………………………………………………………………..
…………………………………………………………………………………..
…………………………………………………………………………………..
…………………………………………………………………………………..
……………………………………………………………………………………

10. The time of the day the plant material being used is collected; time of year and stage of development of plant (whether young, mature, etc):
……………………………………………………………………………………
……………………………………………………………………………………

11. Methods of storage and processing (if any):
…………………………………………………………………………………....
……………………………………………………………………………………
……………………………………………………………………………………
……………………………………………………………………………………

12. Community's beliefs about the causes of disease, its symptoms and diagnosis, the stage at which the disease is treated, etc. (where appropriate).
……………………………………………………………………………………..
……………………………………………………………………………………..
……………………………………………………………………………………..
……………………………………………………………………………………..
……………………………………………………………………………………..

13. Adjunct therapy used (if any):
……………………………………………………………………………………..
……………………………………………………………………………………..
……………………………………………………………………………………..
……………………………………………………………………………………..
……………………………………………………………………………………..

14. Mode and method of administration (dosage/regimen):
…………………………………………………………………………………....
……………………………………………………………………………………
……………………………………………………………………………………
……………………………………………………………………………………
……………………………………………………………………………………
……………………………………………………………………………………
……………………………………………………………………………………
……………………………………………………………………………………
……………………………………………………………………………………

15. Are there any incantations needed? (Yes or No). If yes, write down the incantation below or at the back of the sheet.

16. Other plants or ingredients with which the plant is used for preparation of medicine:

Name (Genus, Specie, Family)	Local Name	Part Used

a. ……………………………………………………………………………….

b. ………………………………………………………………………………..

c. ………………………………………………………………………………...

17. Brief botanical description of plant (including habitat and location where collection was made; indicate color of the flower, features of the fruit and diagnostic and distinguishing morphological features):
………………………………………………………………………………..
………………………………………………………………………………..
………………………………………………………………………………..
………………………………………………………………………………..
………………………………………………………………………………..
………………………………………………………………………………..
………………………………………………………………………………..
………………………………………………………………………………..
………………………………………………………………………………..
………………………………………………………………………………..
………………………………………………………………………………..
………………………………………………………………………………..
………………………………………………………………………………..
………………………………………………………………………………..
………………………………………………………………………………..
………………………………………………………………………………..
………………………………………………………………………………..
………………………………………………………………………………..

18. Name and address of person (Herbalist or Supplier) giving this information about the medicinal value of this plant:
………………………………………………………………………………..
……………………………………………………………………………….....
………………………………………………………………………………..
………………………………………………………………………………..

19. Any other remarks (please indicate below any special information indicated by donor of information e.g. plant to be collected at specific time of day or young part of plant to be used; claims of its efficacy; information on toxicity; etc.)
………………………………………………………………………………..
……………………………………………………………………………….....
………………………………………………………………………………..
………………………………………………………………………………..

20. Name and address of collector/recorder of this information:
………………………………………………………………………………..
……………………………………………………………………………….....

21. Name and address of botanist who identified the plant:
………………………………………………………………………………………..
……………………………………………………………………………….....
………………………………………………………………………………………..
………………………………………………………………………………………..

22. Name and address of taxonomist who confirmed identity of the plant:
………………………………………………………………………………………..
……………………………………………………………………………….....
………………………………………………………………………………………..
………………………………………………………………………………………..

23. Remarks/Comments:
………………………………………………………………………………………..
………………………………………………………………………………………..
………………………………………………………………………………………..
………………………………………………………………………………………..
………………………………………………………………………………………..
………………………………………………………………………………………..
………………………………………………………………………………………..
………………………………………………………………………………………..
………………………………………………………………………………………..
………………………………………………………………………………………..
………………………………………………………………………………………..
………………………………………………………………………………………..
………………………………………………………………………………………..
………………………………………………………………………………………..
………………………………………………………………………………………..

INTELLECTUAL PROPERTY RIGHTS STATEMENT*:** ***It is acknowledged that indigenous peoples are the guardians of their customary knowledge. The information provided and contained in this sheet will be fully protected and the intellectual property rights will be vested with those who have provided such information.

pprai/information sheet on medicinal plants/mw
